# Community prevalence of blood-borne viruses (hepatitis B and HIV) in Ireland

**DOI:** 10.1007/s11845-025-03869-9

**Published:** 2025-01-23

**Authors:** P. Aiden McCormick, Marie O’Grady, Paul Holder, Cillian F. De Gascun, John S. Lambert, Orla Crosbie, Susan McKiernan, Maeve Skelly, Garry Courtney, Brian Hennessy, Kevin Walsh, Roisin Twohig, Kate Browne, Tessa O’Gorman, Vivion Crowley, Seán J. Costelloe, Roz O’Byrne, Orla Gildea, Noreen Montgomery

**Affiliations:** 1 National Hepatitis C Treatment Program HSE, Liver Unit, St Vincent’s University Hospital and UCD, Elm Park, Donnybrook, Dublin 4, DO4 T6F4 Ireland; 2 National Hepatitis C Treatment Program, HSE, Dublin, Ireland; 3 National Virus Reference Laboratory, UCD, Dublin, Ireland; 4 Mater and Rotunda Hospitals and UCD, Dublin, Ireland; 5 Cork University Hospital and UCC, Cork, Ireland; 6 St James’s University Hospital, Dublin, Ireland; 7 University Hospital Limerick, Limerick, Ireland; 8 St Luke’s Hospital, Kilkenny, Ireland; 9 University Hospital Waterford, Waterford, Ireland; 10 Sligo University Hospital, Sligo, Ireland; 11 Mater Misericordiae University Hospital, Dublin, Ireland

**Keywords:** Cirrhosis, Hepatitis B, Human immunodeficiency virus, Migration

## Abstract

**Background:**

Chronic infection with hepatitis B virus and HIV causes significant morbidity and mortality. Effective antiviral treatment is available for both. Ireland has historically been considered a low prevalence country. However, with increasing inward migration and diversity, this may be changing.

**Aims:**

The aim of this study was to measure the community prevalence of hepatitis B virus and HIV infections in Irish residents born between the years 1965 and 1985.

**Methods:**

Anonymised residual serum samples from blood tests ordered by community general practitioners and tested in eight general hospital laboratories, spread across Ireland, were analysed for the presence of Hepatitis B surface antigen and antibodies to HIV.

**Results:**

A total of 6080 samples were analysed for hepatitis B surface antigen including 2993 males, 2807 females and 280 samples for which gender was not recorded. HBsAg was detected in 28/6067 samples giving an estimated prevalence of 0.46% (95% CI 0.32–0.67%). Antibodies to HIV were identified in 18/6064 giving an estimated prevalence of 0.33% (95% CI 0.19–0.47%). The prevalence of both hepatitis B and HIV was significantly higher in Cork (Southwest Ireland) than other centres: hepatitis B (12/1050 vs 16/5017, *p* = 0.014) and HIV (11/1049 vs 7/5015, *p* < 0.001).

**Conclusions:**

The prevalence of hepatitis B virus and HIV infections in this cohort appear to be higher than previously estimated. In addition, their prevalence in the Cork area appears particularly high. Whether this represents a true prevalence or a chance finding will require confirmatory studies.

## Introduction

Blood-borne viruses hepatitis B (HBV), hepatitis C and human immunodeficiency virus (HIV) cause serious illness and significant morbidity and mortality. Effective treatment is now widely available for all three viruses. Hepatitis C virus can be eliminated with a short course of oral anti-viral medication. Conversely, while neither chronic hepatitis B nor HIV can be cured, they can be controlled with long-term oral antiviral therapy, thus preventing further end-organ damage. It is also important to diagnose and treat individuals with these viral infections, from a public health perspective, to prevent onward transmission. Accurate information on community prevalence and transmission patterns is essential for designing appropriate screening and treatment strategies. The epidemiology of these infections is likely to change as a result of migration patterns and other social and cultural trends.

We previously reported that the community prevalence of hepatitis C in Ireland was much lower than previously reported, approximately 0.1% compared to previous estimates of about 1% [1]. This study was performed on anonymised routine biochemistry blood samples sent by general practitioners to general hospital laboratories. Individuals born between 1965 and 1985 were included as public health notification data suggested 70% of those infected were in this age cohort. The population prevalence of both hepatitis B and HIV is believed to be low in Ireland at between 0.1 and 0.2% [2, 3]. We now report results for hepatitis B and HIV in the same birth cohort previously studied for hepatitis C.

## Methods

**Fig. 1:**
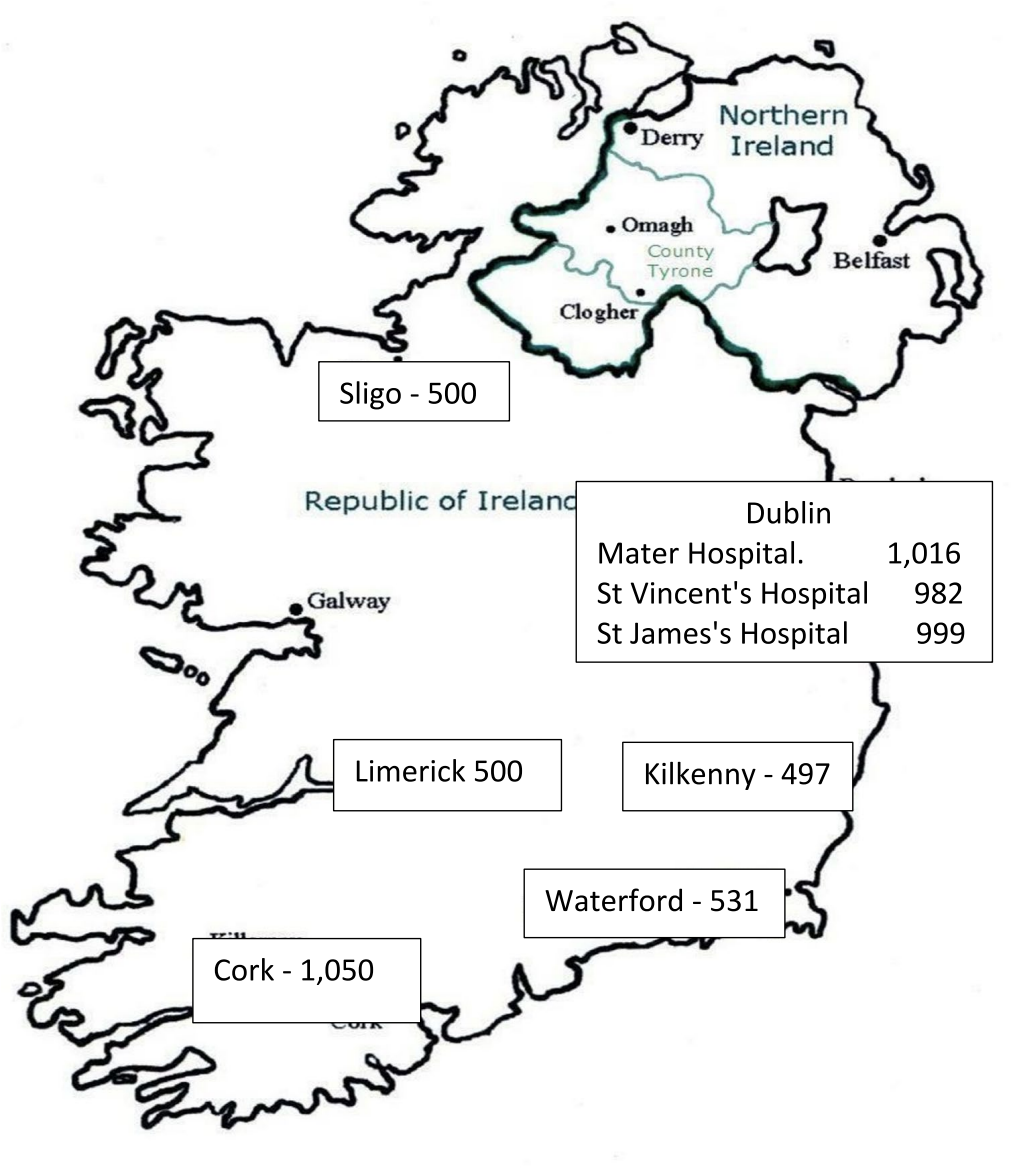
Geographic distribution and number of samples from eight general hospitals participating in the epidemiological study

This study looked at residual serum samples from general practitioner-requested blood tests from eight general hospitals, three of which were in Dublin. These hospitals included St Vincent’s University Hospital, Mater Misericordiae University Hospital, St James’s Hospital, Cork University Hospital, University Hospital Limerick, University Hospital Waterford, St Luke’s Hospital Kilkenny and Sligo University Hospital (Fig. 1). All patients were in the birth cohort (born in years 1965–1985) and the study included equivalent numbers of males and females. Residual samples were anonymised, batched, and sent to the National Virus Reference Laboratory in UCD for analysis. The only information retained on the samples was the sex of the patient. These samples were initially tested for the presence of hepatitis C virus antibody. If positive, the samples were checked for the presence of hepatitis C antigen. Ethical permission was then obtained to test residual samples for HBV and HIV from the research ethics committees in St Vincent’s University Hospital, Mater Misericordiae University Hospital, St James’s Hospital, University Hospital Limerick, Cork University Hospital and University Hospital Waterford. Sligo University Hospital and St Luke’s Hospital Kilkenny accepted the ethics approval from St Vincent’s University Hospital.

## Laboratory methods

HIV status was determined using the Abbott Architect HIV Combo Ag/Ab assay (Abbott Diagnostics, Wiesbaden, Germany). Specimens exceeding the manufacturer’s signal to cut-off (S/Co) ratio of 1.0 were tested using the Biomeriéux HIV DUO Ultra assay (Marcy-L’Etoile, France). Persistently reactive samples were further investigated using the Fujirebio INNO-LIA HIV I/II Score assay (Fujirebio Europe N.V., Ghent, Belgium) to determine the true anti-HIV status of the sample and determine the HIV type.

HBV status was determined initially using the Abbott Architect HBsAg Qualitative II assay (Abbott Diagnostics, Wiesbaden, Germany). Specimens generating a S/Co ≥ 15 were directly investigated for HBV markers using the following assays, Abbott Architect HBeAg (Abbott Diagnostics, Wiesbaden, Germany), Abbott Architect Anti-HBe (Abbott Diagnostics, Wiesbaden, Germany), Abbott Architect anti-HBc IgM (Abbott Diagnostics, Wiesbaden, Germany) and Abbott Architect anti-HBc II (Abbott Diagnostics, Wiesbaden, Germany). Weakly reactive HBsAg samples using the Architect assay (generating HBsAg result above the manufacturer’s S/Co of 1.0 but less than 15.0) or HBeAg and anti-HBe Ab negative samples were further investigated using the Murex HBsAg Confirmatory Version 3 assay (DiaSorin Italia S.p.A UK Branch, Dartford, UK) to determine the true HBsAg status of the sample.

### Statistical analysis

Differences between groups were evaluated using the chi-square (and Fisher’s exact) test using the Prism 10 statistical program (GraphPad Software Inc., California, USA). Confidence intervals (95%) for the estimated prevalence in each sample were calculated using the Clopper-Pearson method [ref1] implemented in the R package epitools [4].

## Results

A total of 6080 samples were available for analysis, including 2993 males, 2807 females and 280 samples for which gender was not recorded. The geographical distribution of samples is shown in Fig. 1. There was insufficient volume to test 13 samples for HBsAg and 16 samples for HIV antibody. The results are summarised in Table 1. HBsAg was identified in 28/6067 samples (18 male, 10 female) giving an estimated prevalence of 0.46% (95% CI 0.32–0.67%). HIV antibody was identified in 18/6064 samples (11 male, 7 female) giving an estimated prevalence of 0.3% (95% CI 0.19–0.47%). Hepatitis C antibodies were detected in 28/6080 (19 male and 9 female), but of these, hepatitis C antigen was detected in only 2/28 (7%) (1 male, 1 female). The geographical distribution of positive samples was non-random. The prevalence of both virus infections was significantly higher in Cork. HBsAg was present in 12/1050 samples (prevalence 1.14%, CI 0.59–2.0%, *p* < 0.01) and HIV antibody in 11/1050 (prevalence 1.05%, CI 0.53–1.87%, *p* < 0.01). None of the samples in any of the hospitals was positive for both HBsAg and HIV antibody. One patient had hepatitis C antibodies and HBsAg (male) and one had hepatitis C antibodies and was anti-HIV positive (female). Both patients were in the Cork cohort. The proportion of females with hepatitis C antibodies was higher in Cork than in the other centres (6 female/3 male vs 3 female/16 male, *p* < 0.05).

**Table 1:** Results of testing for hepatitis B surface antigen (HBsAg), HIV antibodies, hepatitis C antibodies and hepatitis C antigen (HCV Ag) in 6080 community sourced birth cohort samples (born 1965–1985)

Site	Number of samples	Anti-HCV (m/f)	HCV Ag	HBsAg (m/f)	Anti-HIV (m/f)
St Vincent’s Hospital, Dublin	982	2 (2/0)	0	2 (2/0)	0
Mater Hospital, Dublin	1018	3 (2/1)	0	5 (3/2)	4 (2/2)
Limerick	500	2 (1/1)	1 (1/0)	1 (1/0)	0
Waterford	531	1 (1/0)	0	1 (0/1)	0
Sligo	500	2 (2/0)	0	1 (1/0)	1 (1/0)
Kilkenny	500	0	0	0	0
Cork	1050	9* (3/6)	1 (0/1)	12** (9/3)	11** (6/5)
St James’s Hospital, Dublin	999	9 (8/1)	0	6 (3/3)	1 (1/0)
Total	**6.080**	**28 (19/9)**	**2 (1/1)**	**28 (19/9)**	**17 (10/7)**

*p < 0.05. **p < 0.01; Cork prevalence vs other centres combined

## Discussion

In this study, we found the prevalence of hepatitis B surface antigen was 0.46% and the prevalence of HIV antibodies was 0.3% in this community-based cohort of individuals born between 1965 and 1985. These figures appear higher than previously published estimates. As would be expected, approximately two-thirds of those infected were male. A surprise finding was that the prevalence rates of both hepatitis B and HIV were significantly higher in Cork than the other centres combined. We are not aware of a reason for this and it may represent a chance finding. Nevertheless, further investigation is warranted to confirm or refute this finding.

There are limited data on the epidemiology of hepatitis B and HIV in the community in Ireland. A recent publication from the European Centre for Disease Control (ECDC) and Bristol University suggested a population prevalence of 0.21% for HBsAg and a prevalence of 0.26% among prisoners [5]. All pregnant females in Ireland are offered screening for hepatitis B and HIV. The Coombe maternity Hospital in Dublin reported 6974 babies born in 2023. All expectant mothers are checked for hepatitis B, HIV and hepatitis C. Seven were HBsAg positive (0.1%), 18 had HIV (0.26%), 10 had antibodies to hepatitis C but none was PCR positive [6]. The Rotunda Hospital delivered 8442 babies in 2023. Thirty-two had HBsAg (0.38%) and 25 (0.3%) had antibodies to HIV [7]. The prevalence of HBsAg in first time blood donors in Ireland (1999–2022) was very low at 0.009% (monthly donation testing report, UK Health Security Agency). These figures are for donors born in Ireland or the UK, but would constitute a special population as individuals with risk factors are discouraged from donating.

International protection applicants in Ireland are offered blood-borne virus screening as many are from high prevalence countries. Uptake is variable but the national reception centre for international protection applicants, in Balseskin, Dublin, reported that of those tested in 2023, 2.2% were HBsAg positive, 4.3% were HIV positive and 0.3% had chronic hepatitis C infection [8]. The UNAIDS program estimates the prevalence of HIV infection in the general adult population in Ireland in 2023 was 0.3% [9]. All pregnant females are offered antenatal testing for HIV. The national prevalence for 2023 was 0.2% [10]. Only 14% of these were new diagnoses suggesting high rates of previous diagnosis, awareness and treatment in this cohort.

The strengths of this study are that it was community based, with a wide geographical spread and was not targeted at higher risk groups. The major limitation is that the samples were anonymised so we have no clinical information about the infected individuals. It is possible that chance over-representation from higher risk groups may have skewed the results, particularly with regard to Cork, in the southwest of the country.

This study suggests that the epidemiology of hepatitis B and HIV infections is changing in Ireland and that the community prevalence of both is higher than previously estimated. This has implications for screening and service provision. It highlights the importance of up-to-date epidemiological studies, particularly in societies affected by large-scale migration and social changes.

## References

[1] McCormick PA, O’Grady M, De Gascun CF and others (2024) Hepatitis C community prevalence is over-estimated: a prospective, birth cohort study. Ir J Med Sci 193:1257–1260 38285072 10.1007/s11845-023-03604-2 PMC11128382

[2] Nardone A, Anastassopoulou CG, Theeten H and others (2009) A comparison of hepatitis B seroepidemiology in ten European countries. Epidemiol Infect 137:961–969 19102797 10.1017/S0950268808001672

[3] Tuite H, Horgan M, Mallon PW and others (2015) Patients accessing ambulatory care for HIV-infection: epidemiology and prevalence assessment. Ir Med J 108:199–202 26349347

[4] Aragon T (2020) Epitoole: epidemiology tools. R package version 0.5–10.1. http://CRAN.R-project.org/package=epitools

[5] Trickey A, Bivegete S, Duffell E and others (2023) Estimating hepatitis B virus prevalence among key population groups for European Union and European Economic Area countries and the United Kingdom: a modelling study. BMC Infect Dis 23:457 37430220 10.1186/s12879-023-08433-3 PMC10331985

[6] The Coombe Hospital. Annual Report 2023. Available at: https://www.coombe.ie/annual-report. (Accessed 31/12/2024)

[7] Rotunda Hospital Dublin Annual Report 2023. Available at: https://rotunda.ie/rotunda-hospital-annual-report-2023. (Accessed 31/12/2024)

[8] (2023) Health Screening, National Reception Centre, Balseskin. Annual Report. Health Service Executive

[9] UNAIDS HIV and AIDS estimates for Ireland 2023. Available at https://www.unaids.org/en/regionscountries/countries/ireland. (Accessed 31/12/2024)

[10] Antenatal HIV testing in Ireland. Health Protection Surveillance Centre. Available at https://www.hpsc.ie/a-z/hivandaids/antenatalhivtesting/. (Accessed 24/12/2024)

